# Protein Catabolism and High Lipid Metabolism Associated with Long-Distance Exercise Are Revealed by Plasma NMR Metabolomics in Endurance Horses

**DOI:** 10.1371/journal.pone.0090730

**Published:** 2014-03-21

**Authors:** Laurence Le Moyec, Céline Robert, Mohamed N. Triba, Véronique L. Billat, Xavier Mata, Laurent Schibler, Eric Barrey

**Affiliations:** 1 Unité de Biologie Intégrative et Adaptation à l'Exercice, Université d'Evry Val D'Essonne-Inserm, Evry, France; 2 Génétique Animale et Biologie Intégrative, INRA, Jouy-en-Josas, France; 3 Chimie Structures Propriétés de Biomatériaux et d'Agents Thérapeutiques, Université Paris 13 Sorbonne Paris Cité-CNRS, Bobigny, France; 4 Ecole Nationale Vétérinaire d'Alfort, Université Paris Est, Maisons-Alfort, France; National Research Council of Italy, Italy

## Abstract

During long distance endurance races, horses undergo high physiological and metabolic stresses. The adaptation processes involve the modulation of the energetic pathways in order to meet the energy demand. The aims were to evaluate the effects of long endurance exercise on the plasma metabolomic profiles and to investigate the relationships with the individual horse performances. The metabolomic profiles of the horses were analyzed using the non–dedicated methodology, NMR spectroscopy and statistical multivariate analysis. The advantage of this method is to investigate several metabolomic pathways at the same time in a single sample. The plasmas were obtained before exercise (BE) and post exercise (PE) from 69 horses competing in three endurance races at national level (130–160 km). Biochemical assays were also performed on the samples taken at PE. The proton NMR spectra were compared using the supervised orthogonal projection on latent structure method according to several factors. Among these factors, the race location was not significant whereas the effect of the race exercise (sample BE vs PE of same horse) was highly discriminating. This result was confirmed by the projection of unpaired samples (only BE or PE sample of different horses). The metabolomic profiles proved that protein, energetic and lipid metabolisms as well as glycoproteins content are highly affected by the long endurance exercise. The BE samples from finisher horses could be discriminated according to the racing speed based on their metabolomic lipid content. The PE samples could be discriminated according to the horse ranking position at the end of the race with lactate as unique correlated metabolite. As a conclusion, the metabolomic profiles of plasmas taken before and after the race provided a better understanding of the high energy demand and protein catabolism pathway that could expose the horses to metabolic disorders.

## Introduction

During long endurance exercises, horses are undergoing a metabolic stress associated with thermolysis, electrolytic loss, physiologic and energetic adaptations. Many factors including diet, training or hormonal system may interfere with these biological adaptations. The massive and extreme conditions encountered in endurance races are expected to involve several metabolic pathways concerning several organ systems [1], [2]. Long exercise produces many catabolic products coming from energetic metabolism, haemolysis, partial rhabdomyolysis, hepatic and renal metabolism that should be cleared in order to maintain the general homeostasis compatible with endurance exercise continuation.

Compared with more conventional techniques, metabolomics performs a global, non-dedicated analysis of the metabolites contained in a biofluid, a tissue or an organ. Results may be achieved by the combined use of a multiparametric analytical technique, most commonly mass spectrometry (MS) or nuclear magnetic resonance spectrometry (NMR), a multivariate statistical analysis such as principal component analysis (PCA) or projection on latent structure (PLS) analysis. Consequently, metabolomics is well adapted to simultaneously investigate most of the metabolic modifications that occur during endurance races and may be observed at the plasma level. It has been widely demonstrated in human and animals such as rodents that metabolome is highly modified by physical exercise [3], [4]. The effects of long endurance races in horses have been repeatedly investigated from the biochemical point of view but never by using metabolomics. In the field of equine physiology and pathology, NMR metabolomics has only been applied to study laminitis [5] and the response to glucose overload [6].

Identification of metabolomic profiles associated with exercise and related stress, would achieve a better understanding of the great energetic demand resulting from extreme exercise like 160 km endurance race. The muscular mitochondrial oxidative phosphorylation activity should be maintained at its maximal level during the entire race. Then, the final sprint of about a thousand meters should be performed using glycolytic pathway to increase the muscle power for the fast gallop at the finish line. The NMR metabolomics is able to investigate the distribution of small molecular weight and mobile molecules involved in the energetic metabolic pathway like tricarboxylic acid cycle or lipid beta-oxidation.

During endurance races, horses are examined by veterinarians the day before the race and then every 30–40 km on the race to check the health and exercise adaptation of the horses. Depending on race conditions, about 50% of starts are eliminated for metabolic troubles, lameness or the decisions of the rider. Better understanding of the physiological stress during the race could help to adapt training and race management. In addition, riders are looking for objective criteria to characterize the level of fitness and their horse's chances of success on long-distance races.

In this first NMR metabolomic investigation during endurance races, we first aimed to evidence the metabolic effects of the long exercise. The study was designed to include all participants whatever is their performance, finishers and non-finishers. Comparison of pre- and post-ride metabolomic profiles should provide information about the energetic pathway mobilization and the catabolic metabolism of many molecules in the different tissues.

In a second aim, we assume that NMR metabolomic profiling should give clues to further determine a valuable indicator of the horse ability to perform this high energy demanding effort of endurance races. Therefore, pre-ride and post-ride metabolomic profiles were computed in regard to the individual horse performances.

Briefly, the results of plasma NMR-metabolomic profiles were severely affected by long endurance exercise showing the severe protein catabolism and great involvement of energetic and lipid metabolism. The best performers showed different metabolomic profiles from poor performers mainly based on the plasma lipid content.

## Materials and Methods

### Horses

The study design was approved by the Ethics Committee for the Alfort Veterinary School and the University of Paris-Est under number 12/07/11-1; owner informed consent was obtained before any animal manipulation. Horses were recruited on voluntary basis of the owner on three 160 km endurance races (Table 1). All of them were Arab or half-breed Arabian and aged at least 8 years (mean 10.2±2.0 years). Horses were sampled on two occasions: during the afternoon before the race (BE) and 30 minutes after the end of the race or after disqualification during the race (PE). The races were held according to the International Equestrian Federation (FEI) rules for endurance riding. All of them consisted in 6 phases from 20 to 40 km in length, separated by compulsory halts for veterinary checks (namely vet-gates) followed by recovery period of 40 to 50 minutes. As the samples were obtained during real competition events, the protocol included only general questions about feeding but no control of the diet. However, the usual practice is to feed the horses 2–3 hours before the race starts with traditional pellets of concentrates and to give hay *ad libidum*. No other supplement was used. During the ride at each vet-gate, the horses drank water and eat some hay, which brings electrolytes and allows keeping water in the gut. The average speed during the race of horses sampled at PE ranged from 13.1 to 16.1 km.h^−1^ (mean 15.5 (1.2) km.h^−1^). The ranking of horses qualified after the race varied from 1st to 25th and 19 horses were eliminated for lameness, 3 for metabolic disorder and 4 were retired *i.e.* the rider decided to stop.

**Table 1:** Description of the rides and population included in the study.

Race location	Date	Temperature min-max (°C)	Horse samples		
			Before the exercise (BE)	After the exercise (PE)	Pairs (BE, PE)
1	21/05/2011	13–26	18	8	8
2	12/06/2011	11–14	7	15	6
3	02/09/2011	19–28	4	2	
TOTAL			29	26	28

### Samples

Blood was collected with minimal restraint from a jugular vein into commercial evacuated tubes. Blood was collected on sodium fluoride and oxalate in order to inhibit further glycolysis that may increase the lactate levels after sampling. Blood was left to decant at 4°C before plasma was separated from the pellet; then plasma was stored at −80°C until NMR analysis. For biochemical analysis, blood was collected on dry tubes. After clotting, serum tubes were centrifugated and harvested serum was stored at 4°C until analysis which occurred within 48 hours.

After exclusion of horses because of interfering pathologies related to the long exercise (rhabdomyolysis in 2 cases) and non-participating horses (1 case) the total number of analyzed spectra was 83 (Table 2). Among these spectra, 14 pairs collected before (BE) and after (PE) the race were obtained from the same horse in the pair (paired set). The 55 other samples were collected before the race (BE) for 29 of them and after the race (BE) for 26 of them (test set).

**Table 2:** Descriptive data of the paired set and the test set.

	Total number of samples	Non-finisher horses	Finisher horses
Paired Set	28	3	11
Average Speed (km.h^−1^)		16.1(6.1)	15.7 (1.1)
Distance (km)		133, 133, 120	160
Test set			
BE	29	18	11
Average speed (km.h^−1^)		15.8 (0.7)	16.2 (1.7)
Distance (km)		99.2 (33.5)	130 (15)
PE	26	5	21
Average speed (km.h^−1^)		16 (5)	16 (1)
Distance (km)		122.5 (57)	160

Average speed and distance are given as mean values (standard deviations) calculated for the designated group of horses.

The 43 samples collected at BE included 18 finishers and the mean speed could be evaluated for 42 horses. The 40 samples collected at PE included 31 finishers and mean speed could be collected for the 40 horses.

### NMR spectroscopy

The plasmas were thawed at room temperature. In the 5 mm NMR tubes, 600 µL of plasma was added with 100 µL deuterium oxide for field locking. The proton spectra were acquired at 500 MHz on a Bruker Avance II spectrometer with a 5 mm reversed QXI Z gradient high resolution Bruker probe. The temperature was 297 K. The FIDs were acquired using the noesy1D sequence for water suppression including a pre-acquisition delay of 2 s, a 100 ms mixing time and a 90° pulse. The FIDs were collected on 32 K complex points for a spectral window of 6000 Hz and 64 transients after four silent scans. The use of a noesy1D sequence preserves the lipid resonance integrity. The FIDs were processed with the NMRpipe software. The Fourier transform was performed with an exponential function producing a 0.3 Hz line broadening. Spectra were phased and the baseline correction was performed with three points at 0 ppm, 5 and 9 ppm. Each spectrum was calibrated using acetic acid signal at 1.92 ppm. The spectral region between 0 ppm and 9.5 ppm was divided into 9500 spectral regions of 0.001 ppm width called buckets using a personal program with R software. Each bucket is labeled with its median chemical shift value. Water region, between 4.6 and 5 ppm, was excluded. The buckets intensities were normalized using the quantile normalization as described and used previously [7], [8] in order to obtain the X matrix for statistical analysis. The quantile normalization was used to correct the concentration effects due to possible dehydration after the race. Unit variance scaling was performed on all variables before statistical multivariate analysis.

### Biochemical assays

The sera of horses taken after the race (PE) were assayed on a RX Imola analyzer for biochemical values of total bilirubin and conjugated bilirubin, total proteins, creatinine, creatine kinase, aspartate amino transferase (ASAT), gamma glutamyltransferase (GGT) and serum amyloid A (SAA). Blood collected on EDTA was used to measure packed cell volume (PCV) after centrifugation. The effect of the race location was assessed using the ANOVA test and the effect of speed by linear correlation analysis. Both were performed under Excel and a P<0.05 was considered as significant.

### Statistical analysis

A principal component analysis (PCA) was first performed to detect any group separation based on NMR signal variability. This method also enabled detection of any outliers, defined as observations located outside the 95% confidence region of the model.

The aim of the supervised multivariate statistical analyze is to identify differences between samples spectra depending on external factors. These factors were the race location, the blood sampling time (BE/PE), the performance (average speed, ranking) and biochemical parameters. Those factors were encoded in a so-called Y matrix correlated through the Orthogonal projection to latent-structure (OPLS) method to the X matrix. Compared to the classical projection of latent-structure analysis (PLS), this method allowed improved interpretation of the spectroscopic variations between the groups, by removing information that had no impact on the differences. PCA and OPLS analyses were performed using an in-house Matlab code (Mathworks, Natick, MA) based on the Trygg and Wold method [9] as previously described [10].

The parameter quality for the OPLS model was assessed by calculation of R2Yand Q2Y. The R2Y represents the explained variance of the Y matrix. The Q2Y, computed with the “leave-one-out” method, estimates the predictability of the model. R2Y = 1 indicates perfect description of the data by the model, whereas Q2Y = 1 indicates perfect predictability.

For internal validation of the OPLS models, a permutation test (999 permutations) was performed. This evaluated whether the OPLS models, built with the groups, was significantly better than any other OPLS model obtained by randomly permuting the original group attributes.

A score plot illustrated the results. Each points in the score-plot represents the projection of an NMR spectrum (and thus a horse sample) on the predictive (Tpred, horizontal axis) and the first orthogonal component of the model (Torth, vertical axis). The loading plot represents the covariance between the Y-response matrix and the signal intensity of the various spectral domains. Colors were also used in the loading plot depending of the R value associated with the correlation between the corresponding bucket intensity and Y variable. The metabolites were considered as discriminating metabolites when corresponding to the buckets with R over or equal to 0.5 and a p value lower or equal to 10^−4^.

To compare the spectra of plasma sampled at BE and PE, the model was computed with paired samples taken from the same horses (paired set). Then samples taken at BE or PE from different horses (test set) were used to test the ability of the model to discriminate between BE and PE cases. To evaluate the ability of the OPLS latent variable Tpred to correctly classify the test set, a receiver-operator characteristic (ROC) curve was used. The area under the ROC curve (AUROC) was computed. A perfect discrimination corresponded to an AUROC equal to 1.

## Results

**Figure 1:**
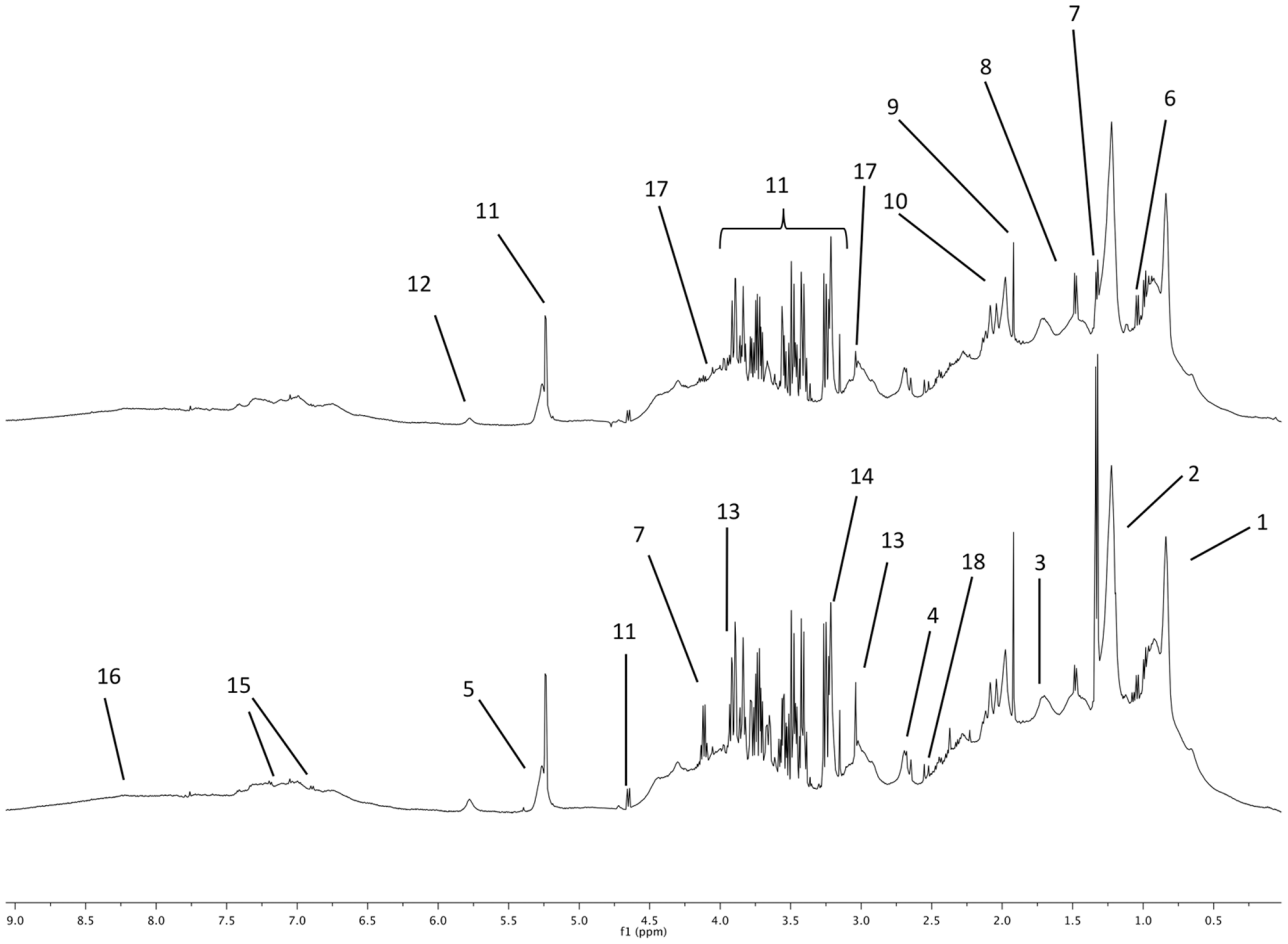
Proton 1D NMR spectra of horse plasma sampled before the race (top spectrum) and after the race (bottom spectrum). The main metabolites are labeled as follows. 1 to 5: lipid fatty acid moieties, 1: methyl; 2: methylene, 3: methylene β ester, 4: methylene α ester, 5: acyl, 6: Branched chain amino acids (valine, leucine, isoleucine), 7: lactate, 8: alanine, 9: acetate, 10: N-acetyl moieties (glycoproteins), 11: glucose, 12: urea, 13: creatine 14: phosphocholine, 15: tyrosine, 16: exchangeable proton from carboxylic moieties, 17: creatinine, 18 : citrate. (The labels of metabolites appearing in both spectra are not repeated).

The NMR spectra obtained at BE and PE are plotted with two examples in figure 1 with the spectral assignments to metabolites. These spectra were similar to horses plasma spectra reported previously [5].

### Metabolomic profile related to the race location

In order to evaluate the effects of external conditions (Table 1), models were computed from plasma NMR spectra of horses at BE and at PE according to race location. The first model computed with BE plasma spectra was not able to discriminate samples according to the race location. The second model computed with PE sample spectra could discriminate sample spectra taken at Race 1 from those taken at Race 2 with a lower predictive coefficient (Q2 = 0.224) than the following classifications and the only discriminating metabolite (with R = 0.5) was citrate.

### Metabolomic profile in plasma before and after the race

**Figure 2:**
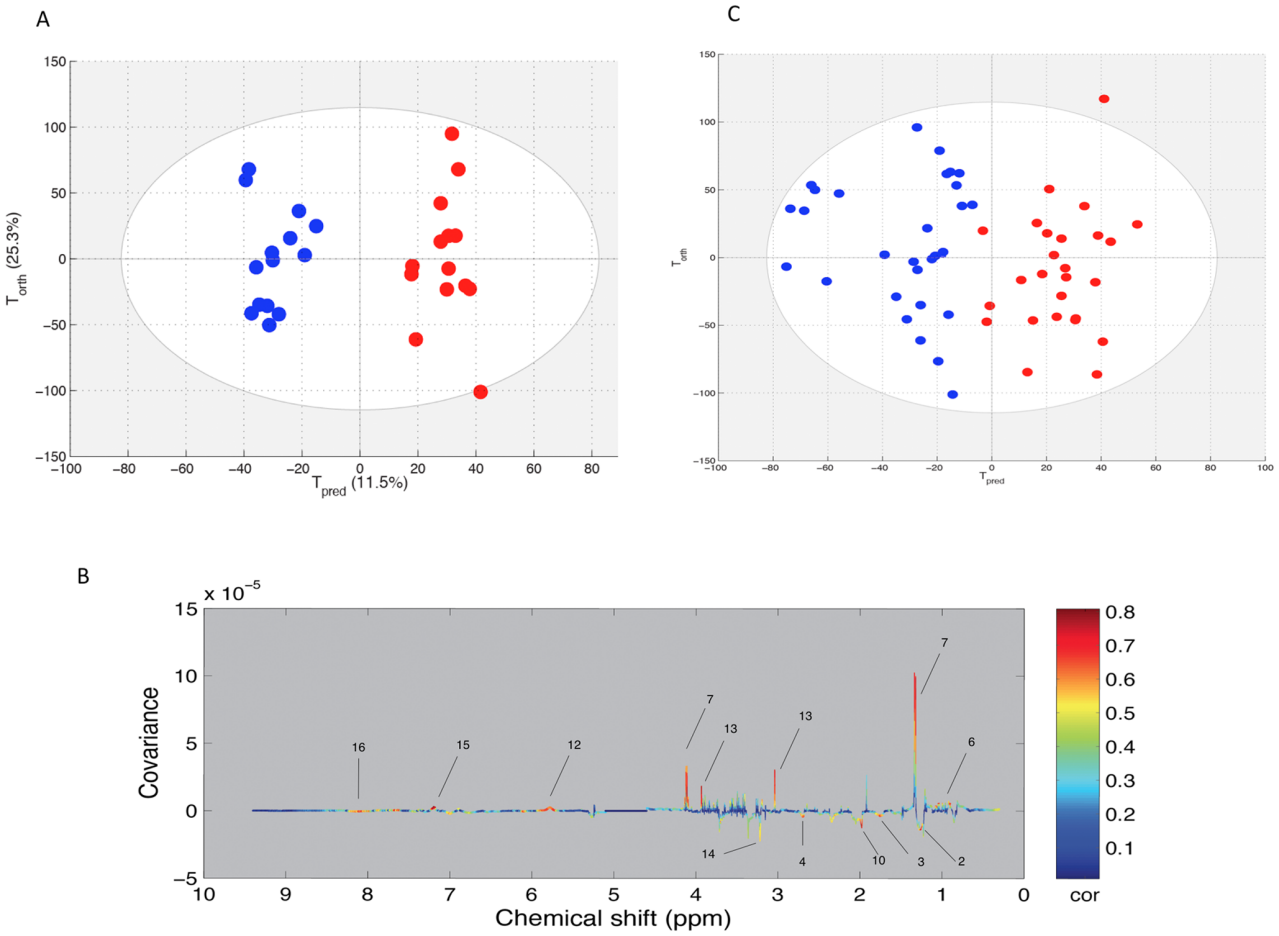
A: Score plot of the OPLS model computed with pre-exercise (BE) and post-exercise (PE) samples from the same horse. Tpred represents the predictive axis and Torth, the orthogonal axis. Each dot corresponds to a spectrum, colored in blue for BE and red for PE. B: Loading plot of the score plot predictive axis. The metabolite correlations are represented by the color scale. Positive signals correspond to metabolites present at increased concentrations at PE. Conversely, negative signals correspond to metabolites present at increased concentrations at BE. The buckets are labeled according to metabolite assignments of figure 1. C: projection of the unpaired sample spectra on the model A. Each new spectrum was projected in the score plot using the previously constructed model to enable prediction of BE or PE spectra.

The PCA model obtained with the paired set did not show any outlier samples and could not discriminate the spectra of BE horse plasma from those of the PE plasma. Consequently, the OPLS model was computed with the 14 samples pairs of the paired set (Table 2) in order to discriminate the metabolomic profiles before and after the race. The score plot of the model is presented in the figure 2A. Good statistical performances were obtained for this model as the R2Y = 0.942 and Q2Y = 0.845. Several variables were highly correlated to the sampling time (BE or PE). These are represented in the loading plot with the colored scale (figure 2B). A positive intensity corresponds to an increase of the metabolite in the PE group when compared to the BE group. On the opposite, a negative intensity corresponds to a decrease in the PE group when compared to the BE group. Consequently, the race effects on the metabolome were increases of lactate, creatine, urea and several branched chain amino acids such as valine and leucine and aromatic amino acids such as tyrosine. On the other hand, signal arising from fatty acid chains of lipids were decreased. Interestingly a signal in the region of N-acetyl moieties at 1.98 ppm (arising from glycoproteins and/or glucosamine) was decreased after the race.

The 55 reminding samples were used as a test set by projection onto the previous OPLS model. This method assessed the general predictive power of the model. As shown in the figure 2C, the model could predict 100% of BE and PE samples correctly in their respective groups. The AUROC was equal to 1 indicating the perfect prediction of the test set (figure not shown).

### Metabolomic profiles according to the performance

The samples spectra obtained at BE and at PE were analyzed separately in the aim to determine whether the performance could influence the plasma metabolome. Only spectra of the samples from horses that have finished the race were considered in this analysis. This means that disqualified horses were not included in the models as ranking and average speed on the full distance considered as supervising factors were not available for the disqualified horses. In both cases, BE and PE samples, the horses finishing the race could not be differentiated from disqualified horses with an OPLS model.

**Figure 3:**
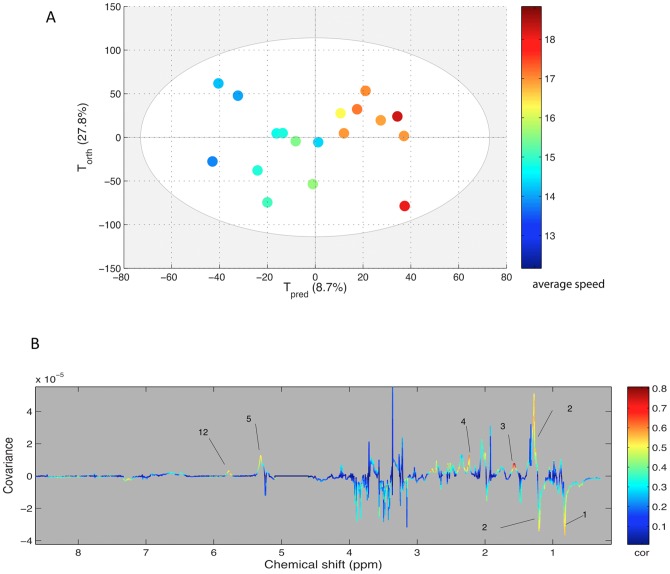
A: Score plot of the OPLS model computed with BE samples from finisher horses classified according to their average speed during the race. Tpred represents the predictive axis and Torth, the orthogonal axis. Each dot corresponds to a spectrum, colored according to their speed values. B: Loading plot of the score plot predictive axis. The metabolite correlations are represented by the color scale. Positive signals correspond to metabolites which concentration increases when speed increases. Conversely, negative signals correspond to metabolites decreasing when speed increases. The buckets are labeled according to metabolite assignments of figure 1.

For spectra obtained at BE from plasma of finisher horses (n = 18), a significant OPLS model could not be computed according to the ranking position. However, when the average speed was used for the same samples, an OPLS model could be computed (figure 3A). The statistical parameters were not very high (R2Y = 0.699, Q2Y = 0.226) but could nevertheless be taken into account as validated through the permutation method. This model is presented here because of the correlated regions of the spectra. As presented in figure 3B, the lipid signals were correlated with the predictive component of the model.

For spectra obtained at PE with plasma of horses finishing the race (n = 32), an OPLS model could be calculated according to the rank (data not shown) with acceptable statistical parameters (Q2Y = 0.363). However, the loading plot showed that the increasing metabolite when rank decreases is essentially lactate. When the spectral regions of lactate were withdrawn from the X matrix, no further model could be computed. With the PE samples and the average speed, no significant model could be computed.

### Metabolomic profile and biochemical data

The mean values of the biochemistry assays are presented in Table 3. Most of the measured values are above the reference values established for horses at rest, but such changes are commonly reported in horses after a 160 km endurance race. Thus, horses show signs of dehydration with increased PCV, total protein and creatinine concentrations. Signs of inflammation, especially in muscle, are evident with high values of CK, ASAT and SAA in all subjects. All horses have a concentration of total bilirubin higher than the reference values, reflecting hemolysis. However, the values of GGT and Conjugated-bilirubin were normal in almost all horses (respectively 42/45 and 32/43), which is in favor of the absence of liver damage.

**Table 3:** Biochemical data obtained on the 45 samples drawn at PE.

Variable	Reference values	Mean values at PE (standard deviation)	Number of cases over the reference values
PCV (l/l)	32–52	52.4 (8.3)	16/37 (43%)
Tot-bilirubin (mg/l)	11.6–21.6	44.6 (14.1)	45 (100%)
Conj-Bilirubin (mg/l)	3.2–5.0	4.51 (0.9)	11/43 (25%)
Tot-proteins (g/l)	50–88	75.2 (6.8)	2 (4%)
Creatinine (mg/l)	9–20	18.0 (4.0)	12 (27%)
Creatine kinase (UI/l 30°C)	150–266	2698 (1771)	45 (100%)
ASAT (UI/l 30°C)	195–280	466 (169)	45 (100%)
GGT (UI/l)	9–20	12.2 (9.1)	3 (7%)
SAA (mg/l)	5–50	807 (515)	42/44 (95%)

Statistical analysis showed a significant low effect of race location with only PCV values being higher in Race 1 and Race 3 when compared to Race 2 (p<0.05). Linear regression analysis showed a significant negative correlation between speed and Conjugated-bilirubin (R = −0.334, p<0.05) and GGT (R = −0.379, p<0.01) values. No other correlation could be established for other biochemical parameters or over the age of horses and their ranking position.

**Figure 4:**
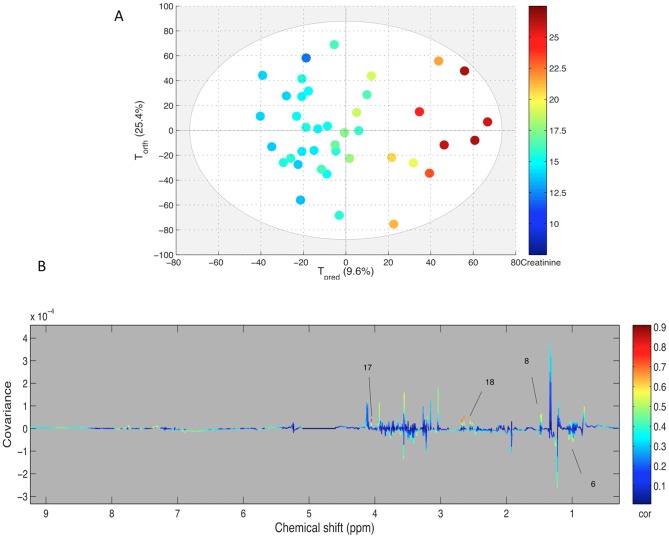
A: Score plot of the OPLS model computed with PE samples according to their creatinine content assessed by biochemistry. Tpred represents the predictive axis and Torth, the orthogonal axis. Each dot corresponds to a spectrum, colored according to their creatininemia. B: Loading plot of the score plot predictive axis. The metabolite correlations are represented by the color scale. Positive signals correspond to metabolites which concentration increases when creatininemia increases. The buckets are labeled according to metabolite assignments of figure 1.

Each biochemical parameter was used as a Y matrix to be correlated in an OPLS model with the NMR spectra obtained from plasma at the same moment. The only significant model was computed with the creatinine values (figure 4). The score plot shows a good discrimination of the creatinine level in plasma spectra with R2Y = 0.886 and Q2Y = 0.597, validated through permutations. The loading plot confirmed the correlation as two main regions, corresponding to citrate (two doublets at 2.5 and 2.7 ppm) and the peak of creatinine methylene at 4.05 ppm, were highly correlated to creatinine. There was no correlation with the methyl peak of creatinine at 3.05 ppm as it was partially overlapped by the high intensity of creatine at 3.04 ppm.

## Discussion

Our results showed that endurance racing consistently modifies the plasma metabolomic profiles of horses after the race (130–160 km) whatever the climatic conditions and the individual performances in the competition. This metabolomic profile is highly dependent on the long exercise effect (sampling time BE *vs.* PE) and the model could be validated with the test set samples from horses with unpaired sample (only BE or PE sample available). The metabolites discriminating the profile before the race from the profile after the race involved in several metabolic pathways such as, protein catabolism, energy metabolism including lipid pathway; metabolites arising from the synovial joint fluid may also be involved in this discrimination.

### Models and performance effect

In the models computed here, the metabolomic profile of horses after different races were included. The different atmospheric and geographic conditions related to the race location could have been a confusing factor impairing the discrimination of the samples taken before and after the race. However, the highly predictive model computed here with horses from three different races showed that the metabolomic profile changes did not depend on race location. Moreover, the validation of the model with data obtained with unpaired plasma confirmed the level of prediction of the metabolomic profile computed.

The same argument may be used about the distance run during the race. In the first computed model, 11 of the horses included had completed the 160 km race and 3 others were non-finisher horses. In the validation test set, the distances reached ranged from less than 100 km up to 160 km. Nevertheless, the final distance run by horses did not interfere with classification of the samples in the PE group as the model correctly classified all PE samples. It has been previously noticed that finishers and non-finisher horses of a 160 km endurance race could be discriminated with biochemical measurements and their body mass loss reflecting their hydration status [11] and that biochemical variables could be used to predict their elimination [12]. In the latter study, the prediction of the completed race was based on biochemical variables standardized with the length of the race and the hydration status. The design of the present study was not addressed to predict the elimination but rather to determine the metabolomic profile modifications after the endurance racing exercise. As both, finishers and non-finishers were included in the paired set and in the test set, the metabolomic profile computed in the present study was not adapted to predict the risk of elimination. In our experimental design, the eliminated horses and placed horses present a similar ‘metabolomic status’ when compared to the metabolomic status before the race.

It may be addressed that the metabolomic profiles determined here were not confirmed by dosages of the same metabolites using another analytical method. However, the metabolite assignments were reported previously [4], [5], [10] and the variations were validated by the projection method. On another hand, the metabolites determined with this NMR metabolomic study are not easily assayed with biochemical methods (N-acetyl moieties or lipids sub-fractions for example).

The model computed with BE plasma and the average speed of finisher horses must be confirmed with a larger number of horses. Nevertheless, it is noteworthy that the metabolites varying with the speed are mainly the lipids. This result may support the importance of nutritional factors and cellulose fermentations producing lipids for the performance during endurance races in order to avoid too much glycolysis metabolism and limit lactic acidosis during the race [13]. Glycolysis metabolism is observed just at the finish line as a higher lactic acid peaks were noticed in the best-ranked performers.

### Protein catabolism

These endurance races are involving the cellular and protein catabolism as signed by the increase of creatine, branched chain amino acids (BCAA) and tyrosine levels in the plasma at PE. In vivo, creatine is found as phosphocreatine in muscle and brain cells. Phosphocreatine is spontaneously transformed into creatine in biofluids and, further, into creatinine eliminated in urine. Consequently, the high level of creatine is a direct consequence of a massive muscle cell lysis and can be considered as a marker of rhabdomyolysis in urine and blood. In NMR spectra, it is possible to differentiate creatine from creatinine as creatinine produces two resonances at 3.05 and 4.05 ppm while creatine's resonances are located at 3.04 and 3.93 ppm. In the loading plot shown in figure 2, the resonances at 3.04 and 3.93 are increased after the race demonstrating that creatine is increased and not creatinine. Besides, the resonance at 4.05 ppm is detectable in spectra but not correlated in the loading plot. Usually, rhabdomyolysis is detected by the increase of creatine kinase (CK) which was elevated in the PE samples collected. In several studies, rhabdomyolysis is also associated with an increased creatinine level in blood as well as uric acid [14] while creatine is never assayed. The present study showed that creatine could be a good sign of the muscle catabolism as it is highly correlated in our model. On the other hand, the creatinine content of serum taken after the race could be used for classification of NMR spectra. With this model, the loading plot shows that the creatinine assays are correlated with the resonance at 4.02 ppm of creatinine and not with the resonances at 3.05 and 3.93 ppm of creatine. Interestingly, the level of citrate in plasma is the most correlated to creatininemia. How citrate and creatinine are linked in the metabolic pathways is not straightforward. Both may result from a catabolic cellular process, plasmatic citrate being the product of mitochondrial lysis occurring with cellular lysis in the liver and muscles. Furthermore, an increased protein catabolism is supported by the increase of BCAA in plasma at PE. BCAA are frequently reported as involved in energy supply in the muscle after their release from the liver [15], [16]. This means that these BCAA reach their target through the general circulation, which might explain the results obtained here. This BCAA mobilization has been related to increased concentration of aromatic amino acids, tyrosine and phenylalanine [17], considered as markers of protein and muscle breakdown [18]. Our results show that tyrosine but not phenylalanine was increased in the plasma of horses after the endurance exercise. Besides, several other amino acids that can be detected in plasma spectra such as alanine, glutamate, lysine, were not participating to the discriminating metabolomic profile. This general profile of severe protein catabolism was in agreement both with biochemistry knowledge in endurance race but also with transcriptomic data describing both severe catabolism and inflammatory status in endurance horses [19], [20].

### Energetics and lipid metabolism

It seems obvious that the aerobic pathway was favored during the long distance endurance races as the glucose level was maintained. However, one of the metabolite with the highest correlation coefficient is lactate. As well known, lactate is the product of an excessive anaerobic metabolism to generate ATP from glucose. The result obtained on PE plasma confirmed this result as the plasma of the best placed horses contained the highest level of lactate with no other metabolite being correlated to their rank. A previous study showed that the measurement of heart rate and lactate level modification after exercise are the simplest methods to evaluate the intensity of exercise in horses [21]. The best horses are fit enough to finish the race at the gallop in a sprint of several hundred meters, using the anaerobic metabolism and thus increasing the level of lactate at PE.

The lipid content of plasma is dramatically reduced by the endurance race. The choline moiety of phospholipids is decreased after the race proving that energy supply from lipids is provided by triglycerides and phospholipids. The fatty acids chain signals are decreased in plasma except for the signal at 5.3 ppm assigned to the olefinic protons (CH = CH) which appear increased after exercise. This long exercise seems to consume preferentially saturated fatty acids preserving the unsaturated fatty acids. The horse lipid metabolism has been investigated only after training protocols and never after an intense endurance race [22]. It has been shown that beside the lactate concentration increase after training sets, several metabolites of lipids including saturated and unsaturated fatty acids were also increased after exercise. In our study, the exercise was very long (about 8 hours) and it seems that the saturated fatty acids were decreased in the blood stream and could have been consumed by heart and muscles. The link between lipid consumption, glucose metabolism and its consequences on lactate level was shown previously [2] with the use of high fat diet. In BE samples the lipid resonances were discriminating metabolites in the model classifying the spectra according to the racing average speed. This result obtained with a limited number of finisher horses needs to be verified in a larger population with a study design dedicated to confirm the importance of the lipid status of horses before the race.

### N-acetyl-glycoproteins origin

When comparing the plasma spectra of horses to those of humans, the main difference is the region between 2 and 2.1 ppm. In human plasma, only one broad resonance is detectable while in horse plasma spectra, three resolved peaks are detected. Similar pattern was published previously [5], [6] and was assigned to N-acetyl moieties of glycoproteins. Many glycoproteins are located at the surface of cells including the red blood cells. Beside glycoproteins, hyaluronic acid contains a high level of N-acetyl glucosamine. Hyaluronic acid is present in the extracellular matrix of most of the tissues and in the joint synovial fluid. The turn-over of hyaluronic acid may be important and increased by tissue injury and inflammation [23]. The presence of NMR detectable N-acetyl functions is related to a relative mobility of the molecules. An increased catabolism of glycoproteins and hyaluronic acid would produce smaller oligomers with higher NMR detectability. Among the spectral regions discriminating BE from PE plasma, the N-acetyl peak with the lowest chemical shift was decreased at PE. This result cannot be explained at this stage of the investigation considering that exercise is expected to increase the catabolism of high molecular weight glycoproteins and hyaluronic acid in muscle and joints during injury or inflammation process. Nevertheless, the higher demand in glycoprotein anabolism may be responsible for this lower N-acetyl signal in the PE plasma spectra. This original result highlights the necessity of further investigations to identify definitively the three N-acetyl glycoprotein NMR resonances of horse plasma spectra in order to elucidate the mechanism of this decrease after endurance exercise.

## Conclusions

The NMR metabolomic investigation of plasma sampled before and after 120–160 km endurance races showed that the metabolomic profile is highly modified by such an extreme endurance exercise in horses. To the best of our knowledge such a metabolomic investigation in endurance exercise is reported here for the first time even considering human endurance athletes. The metabolomic profiles detected in this study demonstrated that adaptation to endurance exercise involved simultaneously energetic, protein catabolism and glycoprotein pathways. The energetic pathway adaptations were revealed by the significant positive correlation between lipid markers and the average speed of the ranked horses. The long exercise stress is mainly revealed by the severe protein catabolism arising from muscle, liver and kidneys tissues undergoing high metabolic disorders. The increase of glycoprotein markers in the blood stream is an unknown phenomenon even in human which should be further investigated. It was assumed to take origin from conjunctive tissues in muscles or cartilages submitted to high mechanical pressure during this long exercise. Furthermore, the metabolomic profiles appear as a relevant and promising method to assess the horse training and metabolic adaptations to endurance exercise.
